# Supplementary material for: Time-Course Transcriptome Profiling Reveals Differential Resistance Responses of Tomato to a Phytotoxic Effector of the Pathogenic Oomycete Phytophthora cactorum
Source: Plants (Basel). 2023 Feb 15;12(4):883. doi: 10.3390/plants12040883 (PMC9964705; doi:10.3390/plants12040883)
Supplement: Supplementary file 1 [file plants-12-00883-s001.zip › Figure S8.pdf]

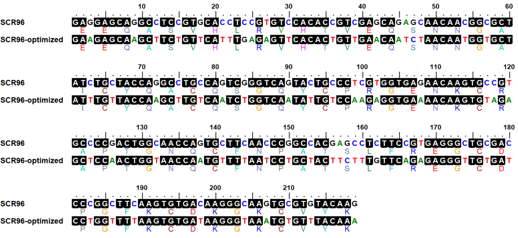
10 20 30 40 50 60
SCR96 GAGGAGCAGGCCTCCGTGCACCTCCGTGTCCACACCGTCGAGCAGAGCAACAACGGCGCT
E E Q A S V H L R V H T V E Q S N N G A
SCR96-optimized GAAGAGCAAGCTTCTGTTCATTTGAGAGTTCACACTGTTGAACAATCTAACAATGGTGCT
E E Q A S V H L R V H T V E Q S N N G A
70 80 90 100 110 120
SCR96 ATCTGCTACCAGGCCTGCCAGTCGGGTCAGTACTGCCCTCGTGGTGAGAACAAGTGCCGT
I C Y Q A C Q S G Q Y C P R G E N K C R
SCR96-optimized ATTTGTTACCAAGCTTGTCAATCTGGTCAATATTGTCCAAGAGGTGAAAACAAGTGTAGA
I C Y Q A C Q S G Q Y C P R G E N K C R
130 140 150 160 170 180
SCR96 GCCCCGACTGGCAACCAGTGCTTCAACCCGGCCACGAGCCTCTTCCGTGAGGGCTGCGAC
A P T G N Q C F N P A T S L F R E G C D
SCR96-optimized GCTCCAACTGGTAACCAATGTTTTAATCCTGCTACTTCTTTGTTCAGAGAGGGTTGTGAT
A P T G N Q C F N P A T S L F R E G C D
190 200 210
SCR96 CCCGGCTTCAAGTGTGACAAGGGCAAGTGCGTGTACAAG
P G F K C D K G K C V Y K
SCR96-optimized CCTGGTTTTAAGTGTGATAAGGGTAAATGTGTTTACAAA
P G F K C D K G K C V Y K
