# Supplementary figures and images for: Time-Course Transcriptome Profiling Reveals Differential Resistance Responses of Tomato to a Phytotoxic Effector of the Pathogenic Oomycete Phytophthora cactorum
Source: Plants (Basel). 2023 Feb 15;12(4):883. doi: 10.3390/plants12040883 (PMC9964705; doi:10.3390/plants12040883)

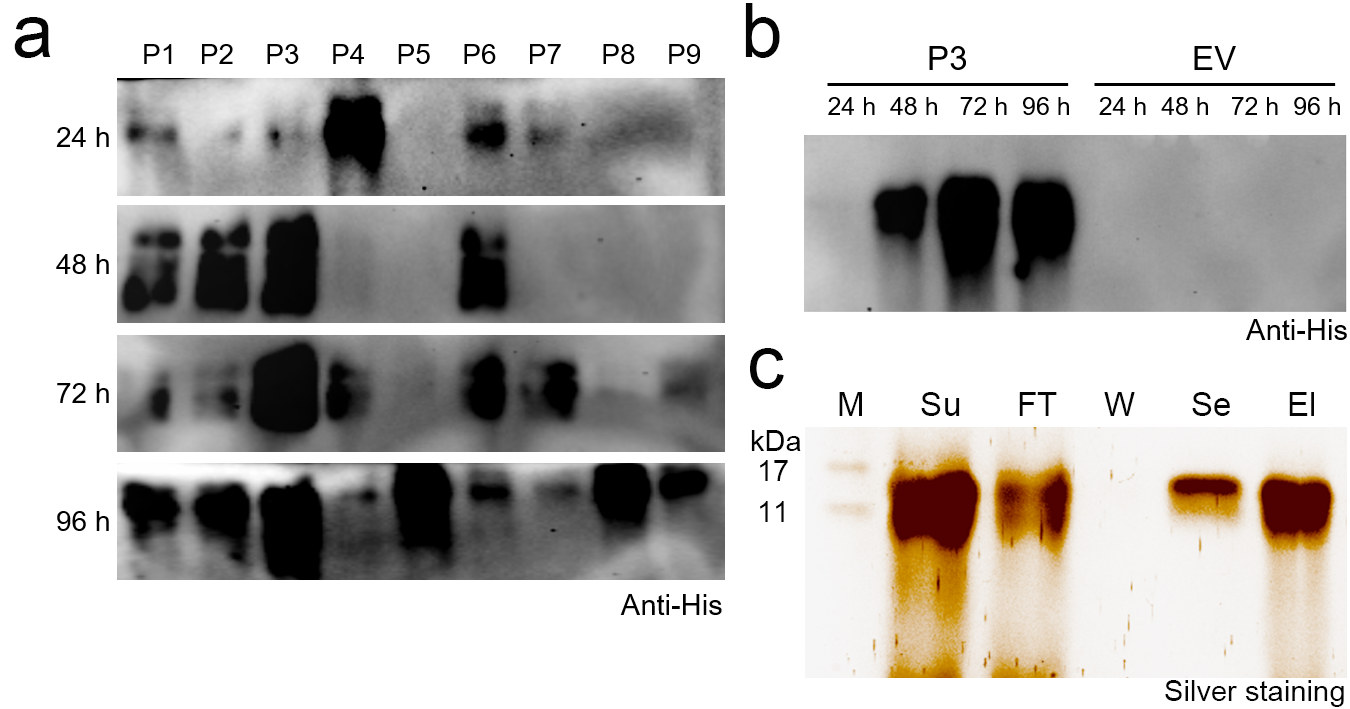

Supplement: Supplementary file 1 [file plants-12-00883-s001.zip › Figure S1.tif]

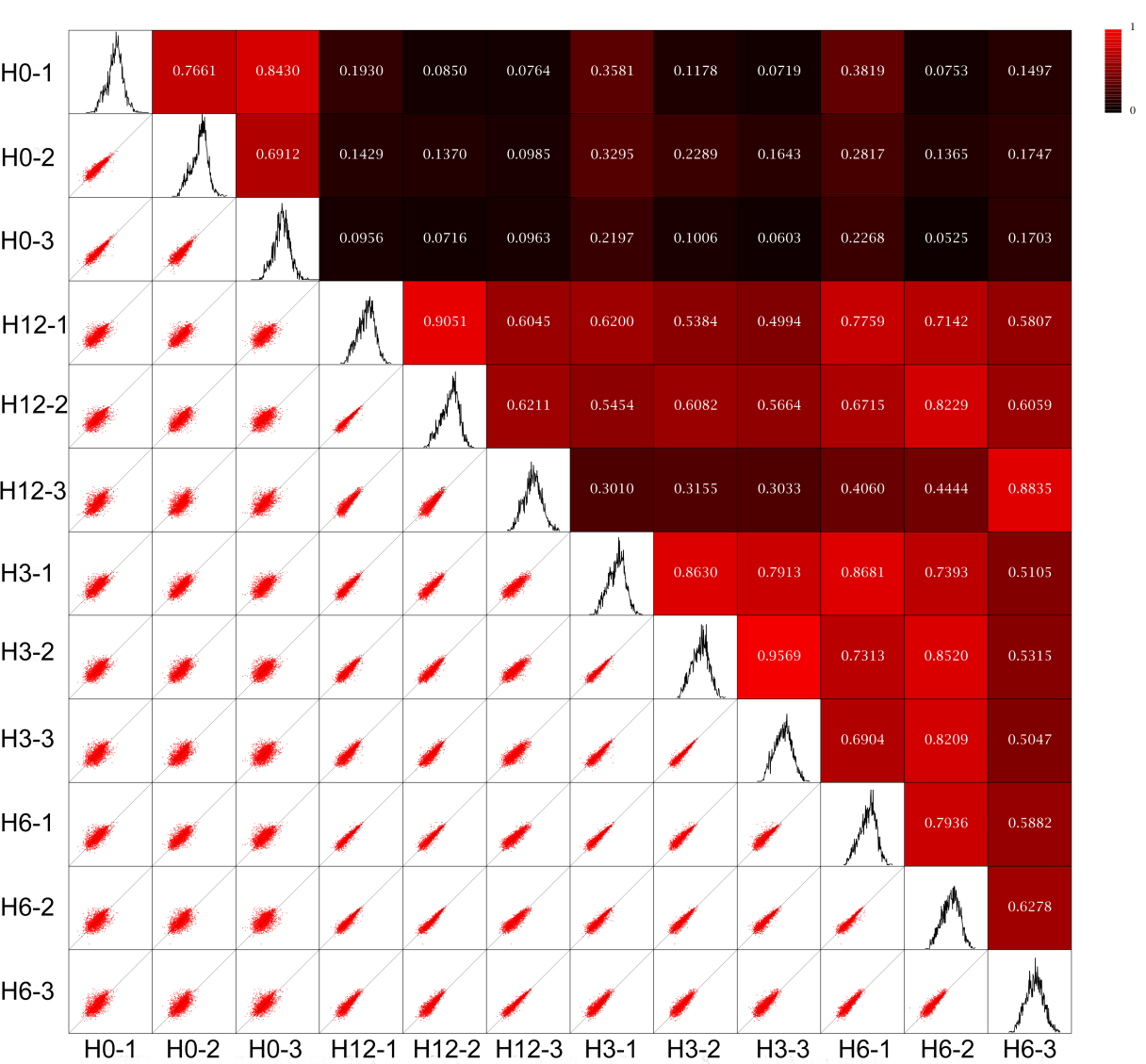

Supplement: Supplementary file 1 [file plants-12-00883-s001.zip › Figure S2.pdf]

# Statistics of Pathway Enrichment

Pathway Name

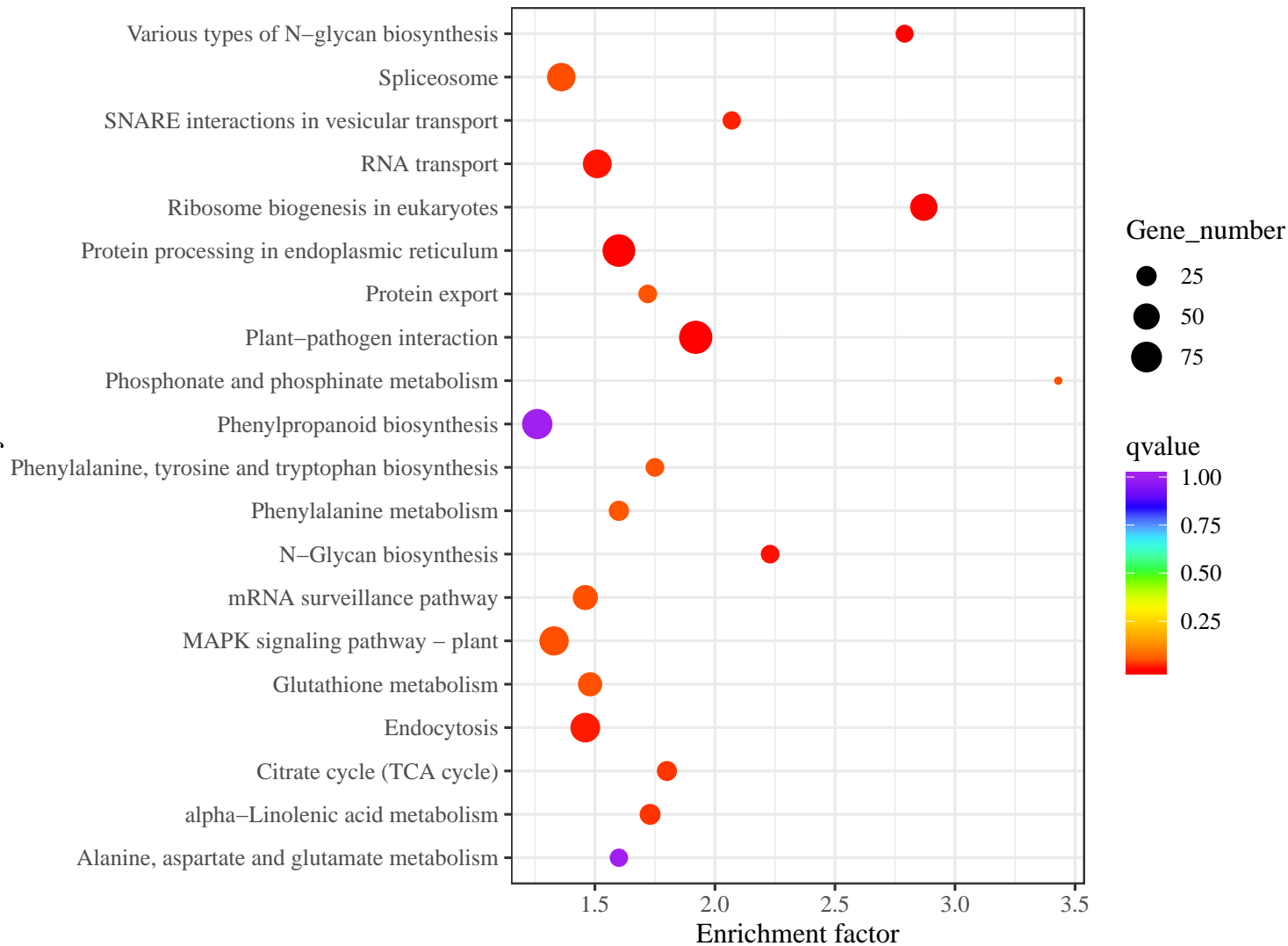

Supplement: Supplementary file 1 [file plants-12-00883-s001.zip › Figure S3.pdf]

***RLKs***

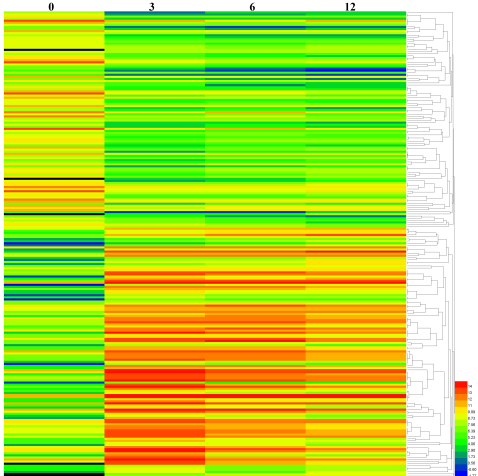

***RLPs***

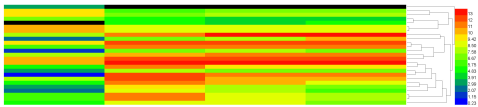

Supplement: Supplementary file 1 [file plants-12-00883-s001.zip › Figure S4.pdf]

**a**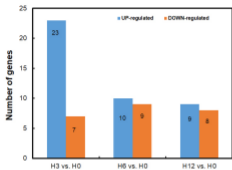**b**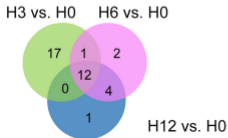**c**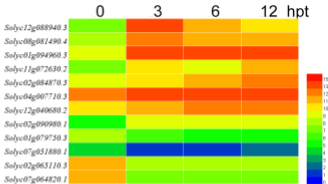

Supplement: Supplementary file 1 [file plants-12-00883-s001.zip › Figure S5.pdf]

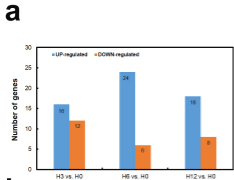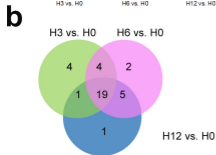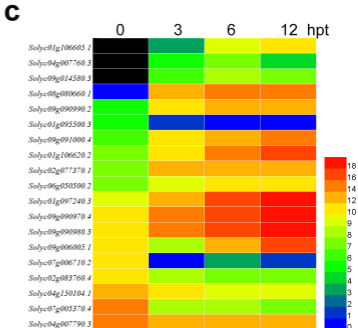

Supplement: Supplementary file 1 [file plants-12-00883-s001.zip › Figure S6.pdf]

0 3 6 12

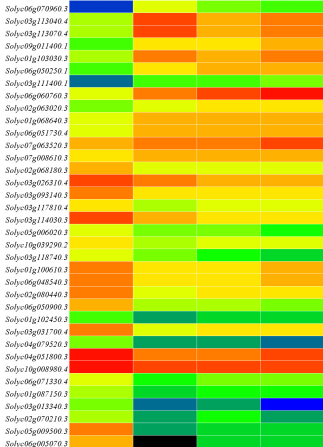

15  
13  
12  
11  
10  
9  
8  
7  
6  
5  
4  
3  
2  
1  
0

Supplement: Supplementary file 1 [file plants-12-00883-s001.zip › Figure S7.pdf]
